# A Comparative Study on the Life-Saving Radioprotective Effects of Vitamins A, E, C and Over-the-Counter Multivitamins

**Published:** 2015-06-01

**Authors:** S. M. J. Mortazavi, S. Rahimi, M. A. Mosleh-Shirazi, M. Arjomandi, A. Soleimani, O. Koohi Hossein-abadi, M. Haghani, M. Alavi

**Affiliations:** 1 Ionizing and Non-ionizing Radiation Protection Research Center (INIRPRC), Shiraz University of Medical Sciences, Shiraz, Iran; 2 Professor of Medical Physics, Medical Physics Department, School of Medicine, Shiraz University of Medical Sciences, Shiraz, Iran; 3 Master Student of Medical Physics, Medical Physics Department, School of Medicine, Shiraz University of Medical Sciences, Shiraz, Iran; 4 Head of Radiotherapy Physics Department, Assistant Professor of Medical Physics, Namazi Teaching Hospital, Shiraz University of Medical Sciences, Shiraz, Iran; 5 Radiologic Technology Student, Radiology Department, School of Paramedical Sciences, Shiraz University of Medical Sciences, Shiraz, Iran; 6 Ph.D Student of Epidemiology, Epidemiology Department, School of Public Health, Shiraz University of Medical Sciences, Shiraz, Iran; 7 Center of comparative and experimental medicine, Shiraz University of Medical Sciences, Shiraz, Iran

**Keywords:** Radioprotective Effects, Vitamin C, Ascorbic Acid, Radiation, Survival

## Abstract

**Introduction:**

Oral intake of vitamins which present antioxidant characteristics can protect living organisms against oxidative damage caused by exposure to ionizing radiation. It was previously reported that administration of high levels of vitamin C can lead to increased DNA damage through production of hydroxyl radicals from hydrogen peroxide by the Fenton reaction. However, our early experiments did not confirm this hypothesis. The main goal of this study was to determine if high doses of Vit C can show life-saving radioprotective effects.

**Materials and Methods:**

Phase I: Seventy two male Balb/c mice weighing 20-25g were randomly divided into six groups of 12 animals each. Group I; Vit E for five days, Groups II and III; Vit C and Vit A. Group 4; all three vitamins. Group V; an over-the-counter multivitamin. Group VI; none of the above. Phase II: 120 male BALB/c mice weighing 20-25g were randomly divided into 12 groups of 10 each. Group I; Vit A for five days. Groups II-IV; Vit C 200 mg/kg, 400 mg/kg, 800 mg/kg, respectively. Group V-VII; Vit E at daily doses of 200 iu/kg, 400 iu/kg, 800 iu/kg, respectively. Group VIII and IX; all three vitamins at low and high doses, respectively. Group X; an over-the-counter multivitamin. Group XI; controls group and Group XII; received pure olive oil. All animals (Phases I and II) were exposed to a lethal dose of gamma rays and the survival rates of the animals were monitored and recorded continuously for 16 days after exposure.

**Results:**

Phase I: 14 days after irradiation the survival rate for control group was 33.33%, while the survival rates for the 1st to 5th groups were 45.45%, 81.81%, 50%, 57.14%, and 9.09% , respectively. Phase II: The survival rates in the control group and the group that only received pure olive oil, were 50% and 60%, respectively. Survival rate in the animals received Vit C at daily doses of 200 mg/kg, 400 mg/kg, 800 mg/kg, were 90%, 90% and 90%, respectively. Log rank (Mantel-Cox) test showed statistically significant differences between the survival rates in control irradiated mice (no vitamins) and mice received Vit C at daily doses of 200 mg/kg (P=0.042), 400 mg/kg (P=0.042) and 800 mg/kg (P=0.042).

**Conclusion:**

Altogether, findings of this study showed that even high doses of Vit C can show life-saving radioprotective effects. The significant radioprotective effect of Vit C at doses used in this study, opens new horizons in developing non-toxic, cost effective, easily available radioprotectors in life-threatening situations such as exposure to lethal doses of ionizing radiation.  The radioprotective effect of Vit A and Vit E seem to be less efficient compared to that of Vit C.

## Introduction

Detrimental effects of low LET radiations are mainly due to generation of free radicals. 

The half life of the free radicals generated by radiation is very short (nanoseconds). Free radicals under aerobic situations cause the formation of reactive oxygen species (ROS)[1]. Free radicals interact with various biological molecules and cause DNA strand breaks, protein oxidation and membrane damage. Oxidative DNA damage is believed to has a critical role in mutagenesis and carcinogenesis[2]. It has been shown that short-lived free radicals significantly modulate the biological effects of radiation such as apoptotic cell death in irradiated cells and tissues[3]. Radioprotectors developed so far are toxic at effective doses and the use of these agents is limited by their side effects and high cost. In this light, exploring effective doses of nontoxic radioprotectors can open new horizons in radiation protection of humans, especially in life threatening situations such as manned deep space missions.  However, to date, no ideal radioprotectors have been developed.

Vitamin C (ascorbic acid, AscH2; ascorbate, AscH−) is a water soluble ketolactone with two ionizable hydroxyl groups[4]. Although plants and the majority of animals synthesize ascorbate from glucose, humans, other primates, guinea-pigs and a few species of fruit-eating bats cannot synthesize ascorbate and need dietary intake of this vitamin[4, 5].

Substantial evidence indicate that vitamin C can serve as an antioxidant to protect DNA damage caused by exposure to ionizing radiation[1]. It has previously been reported that pretreatment of mice with vitamin C can significantly reduce the lethal GI damages induced by ionizing radiation[6]. This study showed that mice pretreated with vitamin C for three days before exposure to lethal whole body radiation of 14Gy and a subsequent bone marrow transplantation (24 h after irradiation), 40% of the mice were rescued. However, post-treatment alone was ineffective in that experiment[6].A more recent study performed by the same group showed that although pretreatment with ascorbic acid alone, engulfment alone, or post-treatment with ascorbic acid alone was not effective in mice irradiated with 13Gy, the combination of pretreatment, engulfment and post-treatment rescued all of the animals (100% survival).This study suggested that post-treatment with ascorbic acid plays a critical role in combination therapy with ascorbic acid[7]. Mortazavi et al. have previously investigated the potential radiation mitigation effect of vitamin C and its possible applications in manned deep space missions. They showed that a single dose of vitamin C can potentially be used up to 24 hours after exposure to reduce the detrimental effects of high levels of ionizing radiation in cases such as the occurrence of currently unpredictable solar particle event[8]. Our current study is an attempt to explore the life-saving radioprotective effects of vitamins A, E, C and an over-the-counter multivitamin.

## Material And Methods

### 1^st^ Experiment

Seventy two male Balb/c mice weighting 20-25g were randomly divided into six groups, each one consisting of 12 animals. The 1st group received Vit E (400 iu/kg) for five days. The animals in the 2nd and 3rd groups received Vit C (400 mg/kg) and Vit A (15000 iu/kg) respectively. Animals in the 4th groups received all three vitamins. Animals in the 5th group received Sanostol, as an over-the-counter multivitamin (0.5 ml/kg). Finally the 6th group received none of the above. All animals were exposed to a lethal dose (LD 50/6) of 8.8 Gy of gamma rays emitted by a Co-60 source on the 6th day at a dose rate of approximately 50 cGy/min. Survival of the animals was monitored continuously for 30 days after irradiation. 

### 2^nd^ Experiment

One hundred twenty male Balb/c mice weighting 20-25g were randomly divided into 12 groups, each one consisting of 10 animals. The 1st group received Vit A(15000iu/kg) for five days. The animals in the 2nd, 3rdand 4thgroups received Vit C at daily doses of 200 mg/kg, 400 mg/kg, 800 mg/kg, respectively. The animals in the 5th, 6th and 7thgroups received Vit E at daily doses of 200 iu/kg, 400 iu/kg, 800 iu/kg, respectively. The animals in the 8th and 9thgroups received all three vitamins at low and high doses, respectively. The 10th group received daily doses of a over-the-counter multivitamin (Nature Made). The 11th served as the control group and finally the 12th group only received pure olive oil. All water insoluble vitamins were dissolved in pure olive oil. All animals were exposed to a lethal dose (LD 50/6) of 8.8Gy of gamma rays emitted by a Co-60 source on the 6th day at a dose rate of approximately 50 cGy/min. Survival of the animals was monitored continuously for 30 days after irradiation. 

## Results

### 1^st^ Experiment

**Figure 1:**
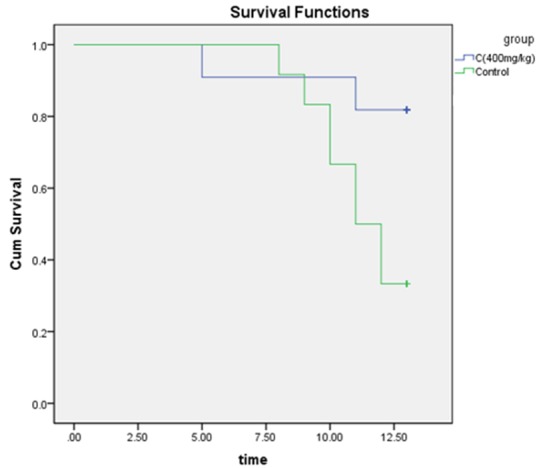
Kaplan-Meier survival plots of the phase I animals pre-treated with vitamins before exposure to a lethal dose (LD) of gamma radiation or only exposed to the lethal dose.

Vitamin C served as the leading efficient radioprotector in this study. Mixture of all three vitamins, vitamin A and vitamin E ranked the 2nd to 4th. Sanostol had no radioprotective effects. Two weeks after irradiation the survival fractionsfor control group was33.33%, while it was 45.45%, 81.81%, 50%, 57.14%, and 9.09% in the 1st to 5thgroups. The mean survival time in control irradiated mice (no vitamins) and mice received Vit C at daily doses of 400 mg/kg were 11.25±0.47 and 12.09±0.70 days, respectively. Log rank (Mantel-Cox) test showed statistically significant differences between the survival rates in control irradiated mice (no vitamins) and mice received Vit C at daily doses of 400 mg/kg (P=0.032). The differences observed between control group and other groups were not statistically significant. Kaplan-Meier survival plot of the animals pre-treated with vitamin C before exposure to a lethal dose (LD) of gamma radiation or only exposed to the lethal dose are shown in Figure 1.

### 2^nd^ Experiment

**Figure 2:**
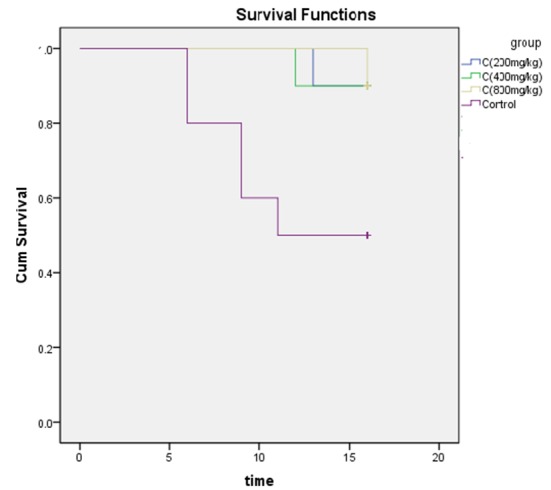
Kaplan-Meier survival plots of the phase II animals pre-treated with vitamins before exposure to a lethal dose (LD) of gamma radiation or only exposed to the lethal dose.

Thirty daysafter irradiation, survival rate in the 1st group that received vitamin A was 100%. Survival rate in the animals of the 2nd, 3rd and 4thgroups that received Vit C at daily doses of 200 mg/kg, 400 mg/kg, 800 mg/kg, were 90%, 90% and 90%, respectively. Survival rate of the animals in the 5th, 6th and 7thgroupsthat received Vit E at daily doses of 200 iu/kg, 400 iu/kg, 800 iu/kg, were 90%, 80% and 100%, respectively. Survival rate of the animals in the 8th and 9th groups that received all three vitamins at low and high doses, were 100% and 100%, respectively. The survival rate in the 10th group that received daily doses of a common over-the-counter multivitamin (Nature Made) was 90%. Finally, survival rate in the 11thgroup which served as the control group and the 12th group that only received pure olive oil, were 50% and 60%, respectively. The mean survival time in control irradiated mice (no vitamins) and mice received Vit C at daily doses of 200, 400 and 800 mg/kg were 12.10±1.31, 15.70±0.29, 15.60±0.38 and 16.00±0.00 days, respectively. On the other hand, the mean survival time in mice received only olive oil, those received an over-the-counter multivitamin and mice received Vit E at daily doses of 200, 400 iu/kg were 13.10±1.18, 15.30±0.66, 15.70±0.29 and 14.90±0.70 days, respectively. Log rank (Mantel-Cox) test showed statistically significant differences between the survival rates in control irradiated mice (no vitamins) and mice received Vit C at daily doses of 200 mg/kg (P=0.042),  400 mg/kg (P=0.042) and 800 mg/kg (P=0.042). This test also showed a statistically significant difference between the survival rates in control irradiated mice (no vitamins) and mice received Vit E at a daily dose of 200 iu/kg (P=0.042). The differences observed between control group and other groups were not statistically significant. Kaplan-Meier survival plot of the animals pre-treated with vitamin C before exposure to a lethal dose (LD) of gamma radiation or only exposed to the lethal dose are shown in Figure 2.

### Pooled data

**Figure 3:**
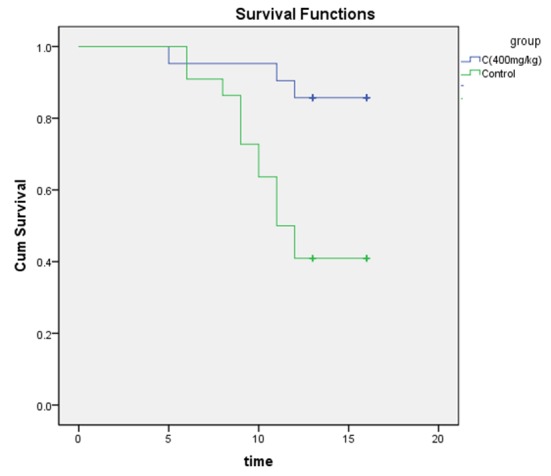
Kaplan-Meier survival plot of the animals pre-treated with vitamin C before exposure to a lethal dose (LD) of gamma radiation or only exposed to the lethal dose in both experiments (pooled data).

After pooling the data obtained in both phases, the mean survival time in control irradiated mice (no vitamins) and mice received Vit C at a daily dose of 400 mg/kg were 12.18±0.75 and 15.05±0.57days, respectively. Log rank (Mantel-Cox) test also showed a statistically significant difference between the survival rates in control irradiated mice (no vitamins) and mice received vitamin C at a daily dose of 400 mg/kg (P=0.002). Kaplan-Meier survival plot of the animals pre-treated with vitamin C before exposure to a lethal dose (LD) of gamma radiation or only exposed to the lethal dose in both experiments (pooled data) are shown in Figure 3.

## Discussion

Findings of this study show that vitamin C serves as the leading efficient radioprotector among vitamins studied. Vitamins A and E were not as effective as vitamin C. Generally, our findings are in line with the results reported recently by other investigators on the role of vitamin C in decreasing damages caused by exposure to ionizing radiation[7, 9-12]. Our findings are not in line with the results of a previously published report which claimed administration of high levels of vitamin C can lead to increased DNA damage through production of hydroxyl radicals from hydrogen peroxide by the Fenton reaction[13]. Our findings are more generally in line with observations of Xiao  et al., who recently investigated the protective role of a lipophilic vitamin C derivative (PlmtVC) on reducing the X-ray radiation-induced damages such as cell death, DNA double-strand breaks, lipid peroxidation, and protein carbonylation.[9]. Invesigators who studied the radiation-induced effect of cytochrome c and vitamin C on the survival of MCF-7 cancer cells grown in aerated media. It has shown that cell survival strongly depends on the action of free radicals produced at the given concentration of the incubated CytC and VitC [10]. It has also been shown recently that all of the mice receiving abdominal radiation at 13 Gy and orally administered ascorbic acid (250 mg/kg/day) for 3 days before exposure, one shot of engulfment (250 mg/kg) at 8 h before exposure, or administered ascorbic acid for seven days after radiation survived (100% survival). Interestingly, none of the control mice survived.  In this light, combination therapy using ascorbic acid consisting of pretreatment, engulfment and post-treatment can rescue all of the mice from lethal effects of the abdominal radiation[7]. Our findings confirm the results obtained in our previous study which investigated the survival rate in animals received vitamin C 1h, 12h and 24h after irradiation.  This study showed that vitamin C can potentially be used up to 24 hours after exposure to high levels of ionizing radiation in life threatening situations such as unpredictable solar particle events. Therefore, we hypothesized that astronauts will have the critical opportunity of evaluating their radiation exposure, before choosing any therapeutic interventions such sd using radiation mitigators[14]. The significant radioprotective effect of vitamin C at doses used in this study opens new horizons in developing non-toxic, cost effective, easily available radioprotectors in life-threatening situations such as exposure to lethal doses of ionizing radiation.
